# Trifurcated Mental Foramina: A Cone-Beam Computed Tomography Incidental Finding During the Implant Treatment Planning

**DOI:** 10.7759/cureus.33828

**Published:** 2023-01-16

**Authors:** Rutvi Vyas, Anita Gohel

**Affiliations:** 1 Oral and Maxillofacial Radiology, University of Florida Health, Gainesville, USA

**Keywords:** anatomical variation, incidental finding, mental foramen, cone beam computed tomography – cbct, anatomy

## Abstract

The mental foramen is a known skull anatomical structure located bilaterally on the mandible along the buccal cortical plate. It is located approximately between the roots of premolars in the anteroposterior dimension, and its supero-inferior level on the alveolar height varies in every individual. The position of the mental foramen is very crucial when surgical interventions are planned in the area. An accessory mental foramina can be very well detected in the three-dimensional (3D) imaging modality, especially in 3D volume rendering images. It can still be appreciated in two-dimensional (2D) imaging modalities such as a panoramic; however, at times it can be confused with periapical pathology, especially in cases where caries are present in the teeth. Three-dimensional imaging modality plays a critical role in identifying such anatomical variation, and hence, it is important to evaluate any surgical site in three dimensions prior to surgical intervention.

## Introduction

The mental foramen is located bilaterally on the mandible along the buccal cortical plate. It is located approximately between the roots of premolars in the anteroposterior dimension, and its supero-inferior level on the alveolar height varies in each individual [1-3]. The mental foramen is generally a singular entity located on each side of the mandible and is the anterior limit of the mandibular canal. It transmits the terminal branch of the inferior alveolar nerve, which is the mental nerve, and its corresponding vessels, which are the mental artery and the mental vein [4]. The mental nerve supplies the lower lip, buccal mucosa, and skin of the chin ventral to the foramen [4].

The position of the mental foramen is very crucial when surgical interventions are planned in the area. In cases of implant planning, the location of the mental foramen is essential, especially when there is alveolar ridge resorption, which may lead to a superior location of the foramen. In cases where osteotomy is planned in the area, it is very critical to evaluate the position of the foramen to avoid injuries to the neurovasculature within the foramen.

An accessory mental foramina can be very well detected in the three-dimensional imaging modality, especially in three-dimensional (3D) volume rendering images. It can be appreciated in two-dimensional (2D) imaging modalities such as a panoramic; however, at times it can be confused with periapical pathology, especially in cases where caries are present in the teeth [5].

In our case report, we present a unique case of a trifurcated mental foramen, which was incidentally detected when evaluating the cone-beam computed tomography (CBCT) scan for implant planning in the area.

## Case presentation

A CBCT scan of a 76-year-old male patient was sent for a radiographic evaluation for implant planning at the edentulous site 19. The CBCT scan had a limited field of view, extending from teeth 18-20. The CBCT scan was acquired using the Carestream (Canada) CS9600 at a 0.1-mm voxel size resolution.

**Figure 1:**
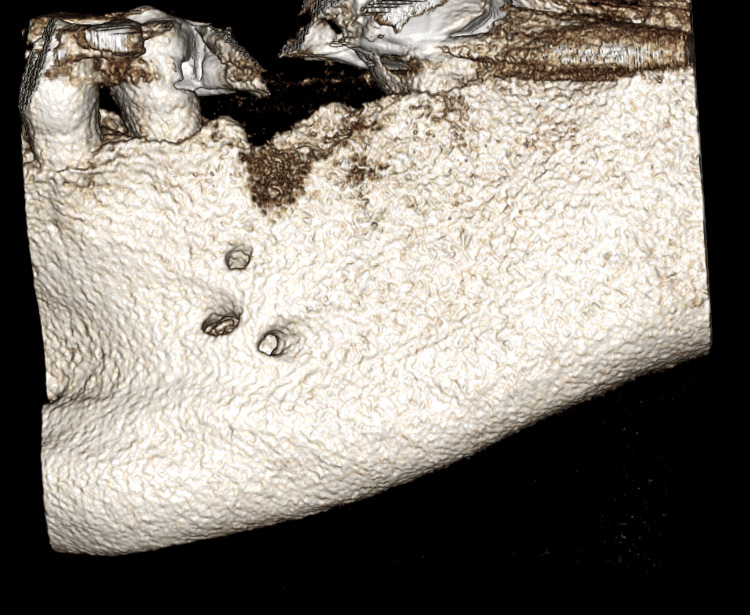
Volumetric reconstruction of CBCT scan showing three mental foramina.

**Figure 2:**
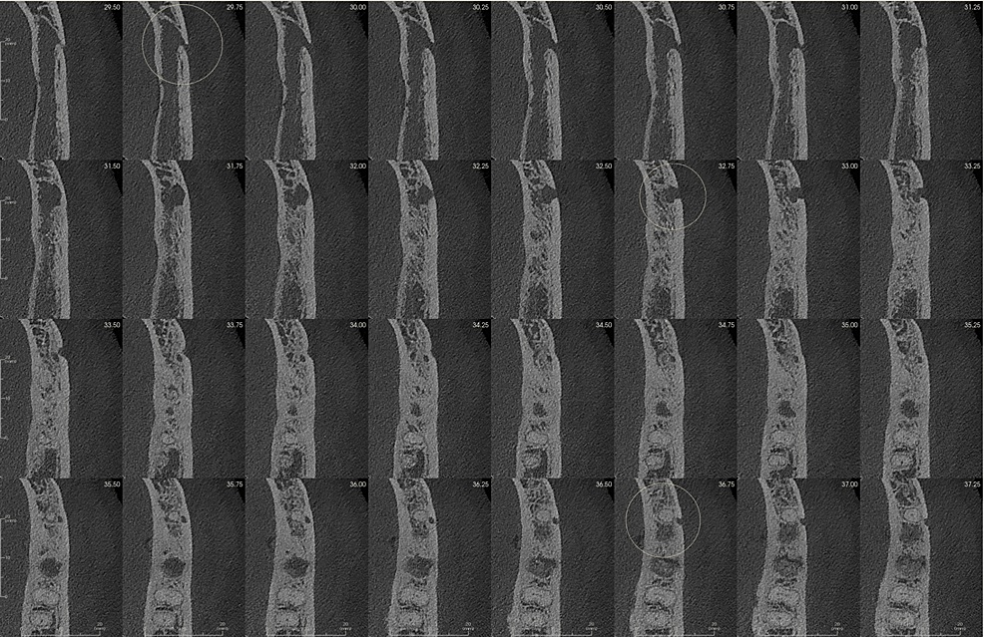
Axial cross-section images at 0.25 mm intervals show the presence of three mental foramina.

**Figure 3:**
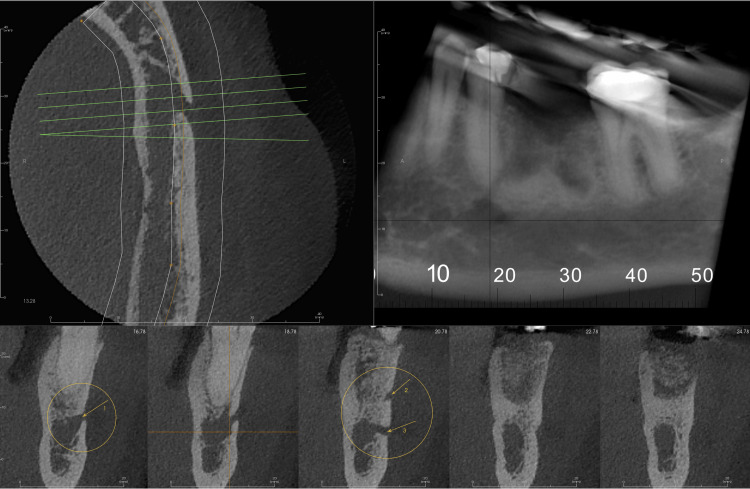
Axial, cropped panoramic reconstruction and multiple cross-sections at 2.0 mm intervals showing the presence of three mental foramina.

Initial evaluation of the overall anatomy was done using 3D volumetric reconstruction, which showed three mental foramina on the captured section of the left body of the mandible (Figure 1). The CBCT scan was further evaluated in the coronal, axial, and sagittal cross-sections; these planar reconstructions showed multiple well-defined oval openings on the buccal cortical plate in continuation with the inferior alveolar canal. These cross-sections were then analyzed in multiple sequential cross-sections for the detailed proximity of these multiple foramina (Figures 2-3).

**Figure 4:**
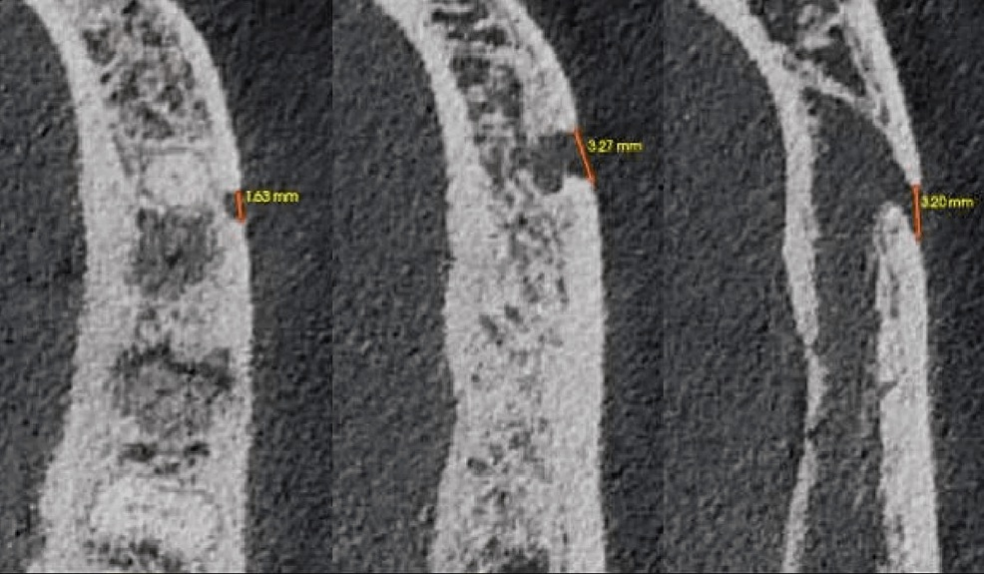
Antero-posterior width of each mental foramen starting from the superior to inferior.

All three foramina were located apically to the root of tooth 20 within a few millimeters of each other (Figure 1). The superior most foramina measured approximately 1.63 mm in the anteroposterior direction, the mid-level foramina measured 3.27 mm in the anteroposterior direction, and the inferior most foramen measured 3.20 mm in the anteroposterior direction (Figure 4).

**Figure 5:**
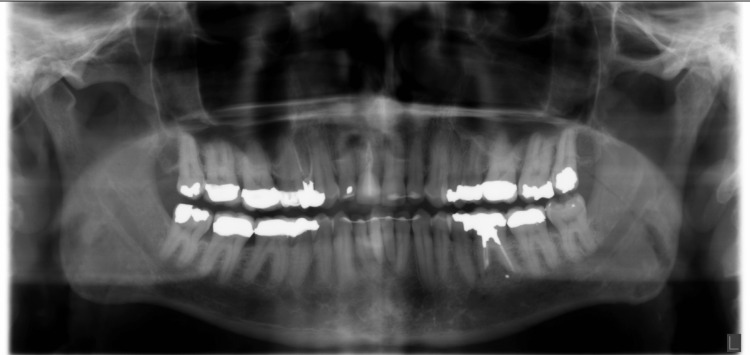
Panoramic radiograph: the three mental foramina are not visualized on the left side.

Additional 2D radiographs, such as panoramic and periapical radiographs, that were available in the patient’s record were evaluated for comparison; these multiple foramina were not appreciated in the 2D radiographs (Figure 5).

## Discussion

Several studies have been published in the literature demonstrating the presence of accessory mental foramina [5-8]. These accessory mental foramina have been coined with several different terminologies in the literature, such as accessory mental foramina, multiple mental foramina, bifurcated or double mental foramina, or even trifurcated mental foramina [4,8-10]. A thorough three-dimensional evaluation of the critical anatomical structures is important whenever any surgical intervention is planned in the area to avoid injury to these structures and subsequent potential complications.

The presence of an accessory mental foramen is considered rare, with its prevalence in the literature showing a varied presentation; it is reported to be between 5% and 30% [11-15].

Although two-dimensional radiographs play a key role in the initial scanning of the area of interest, they lack the actual perception of proximity as well as true identification of the entity in the area. In our case, the multiple mental foramina on the left side were not visualized in the panoramic radiograph. Since the mental foramen opening may be slanted, it may not be clearly visualized due to the superimposition of normal bony trabeculations or distortion of structures due to technique errors [16]. CBCT imaging was able to provide the accurate location of all the foramina and is essential to the optimal planning of implant placement in this region.

The risk of injury to the neurovasculature in the mental foramen may not just be associated with surgical procedures but also with endodontic treatment in the area [17-18]. A CBCT evaluation of the area may be considered in cases where anatomical variation is suspected, either from the 2D radiographs or in cases that have a high potential for anatomical variation. In the current case, because it was a limited field-of-view CBCT scan, we were unable to evaluate the contralateral side for the presence of any accessory mental foramina. In this case, there is an increased possibility of accessory foramina on the right side, and CBCT imaging should be considered when planning any surgical intervention in the area. The risk of neurovascular injury can also be associated with the anesthetic procedure while giving local anesthesia in the area [17].

In our case report, the presence of three mental foramina could have complicated the implant surgery if they were not correctly identified. The proximity of these foramina to the implant site played a critical role in the treatment planning, especially in selecting the size and the angulation of the implant placement.

## Conclusions

Three-dimensional imaging modalities play a critical role in identifying such anatomical variation. Optimal imaging and proper identification of this anatomical variation will provide the correct diagnosis of accessory mental foramina and prevent improper patient care. In order to avoid any complications of regional paresthesia and bleeding, it is crucial to obtain and examine a CBCT scan for accessory foramina during implant placement, apical surgery, or orthognathic surgery in that region.
